# Combination of PURE-DNA extraction and LAMP-DNA amplification methods for accurate malaria diagnosis on dried blood spots

**DOI:** 10.1186/s12936-018-2527-7

**Published:** 2018-10-22

**Authors:** Jeanne Perpétue Vincent, Kanako Komaki-Yasuda, Moritoshi Iwagami, Satoru Kawai, Shigeyuki Kano

**Affiliations:** 1 Department of Tropical Medicine and Malaria, Research Institute, National Center for Global Health and Medicine, Tokyo, 162-8655 Japan; 2 Graduate School of Comprehensive Human Sciences, University of Tsukuba, Ibaraki, 305-8575 Japan; 3 Department of Tropical Medicine and Parasitology, Dokkyo Medical University, Tochigi, 321-0293 Japan; 4 SATREPS Project for Parasitic Diseases, Vientiane, Lao People’s Democratic Republic

**Keywords:** Malaria diagnosis, Procedure for ultra rapid extraction (PURE), Loop-mediated isothermal amplification (LAMP), Dried blood spots (DBS), Nested PCR

## Abstract

**Background:**

Malaria is one of the most important parasitic infectious diseases for which almost half of the world’s population is at risk. Although several diagnostic methods are now available to detect the infection, more sensitive and applicable tests are still required in the field. The loop-mediated isothermal amplification (LAMP) method is a DNA amplification tool in which the DNA amplification can be achieved by incubation at a stable temperature. A malaria detection kit based on this methodology has already been commercialized and is being used in some countries. The kit includes two reaction tubes: one targeting the common *Plasmodium* genus (Pan tube) and the other specifically targeting *Plasmodium falciparum* (Pf tube). In parallel, a simple DNA extraction method, the procedure for ultra rapid extraction (PURE), which can produce a DNA solution suitable for the LAMP reaction without the use of a centrifuge, has also become available. In this study, the sensitivity of the combination of the PURE and LAMP methods (PURE–LAMP) was evaluated with archived dried clinical blood samples of imported malaria cases, including *P. falciparum*, *Plasmodium vivax*, *Plasmodium ovale*, and *Plasmodium malariae*.

**Results:**

Using a nested PCR as the reference, 117 samples including 46 *P. falciparum*, 7 *P. vivax*, 9 *P. ovale*, 4 *P. malariae*, and 51 negative cases were tested. The PURE–LAMP Pan correctly identified 64 of the 66 positives and the 51 negatives. Among the Pan-positive samples 45 *P. falciparum* were also detected with the PURE–LAMP Pf. The PURE–LAMP Pan and PURE–LAMP Pf had respective sensitivities of 96.96% (95% CI 89.47–99.63) and 97.82% (95% CI 88.47–99.94) and common specificity of 1.

**Conclusion:**

The PURE–LAMP system is accurate when used with dried blood spots and extendable to the field.

**Electronic supplementary material:**

The online version of this article (10.1186/s12936-018-2527-7) contains supplementary material, which is available to authorized users.

## Background

As one of the world’s major infectious diseases, malaria totaled around 216 million cases in 2016 [1]. Since the discovery of the parasites in human erythrocytes by Laveran in 1880, the capacity for malaria diagnosis has been paired with the development of biomedical techniques [2–4]. However, the observation of Giemsa-stained parasites under light microscopy remains the gold standard diagnostic technique. It presents various advantages such as allowing estimation of parasitaemia, evaluation of parasite morphology, differentiation between *Plasmodium* species or sometimes with other causes of fever, and economic affordability. Nevertheless, it can only diagnose parasitaemia with about 10–100 parasites/μL, and its sensitivity varies depending on the microscopist’s skill and the quality of the smear [5]. Rapid diagnostic tests (RDT) based on immunochromatography are also commonly used for malaria diagnosis. They are an extremely convenient point-of-care diagnostic method as they require no electrical power, no special skills, and results can be easily obtained within 15 min. Although they perform with high sensitivity on *Plasmodium falciparum* infections of ≥ 100 parasites/μL, they are less accurate for other species. Furthermore, false-positive results are not rare; HRP-2 Pf-specific antigen detection by RDT is useless for the evaluation of recent treatment due to its persistence in the blood for weeks after treatment [6, 7].

In endemic fields, differential diagnosis with other tropical diseases is of great importance; confirmation of the diagnosis before treatment prevents unnecessary exposure to drugs, which can facilitate the rise of drug-resistant parasites. This is also important from the point of view of preventing unnecessary risk of adverse drug reactions and wastefulness. This optimizes treatment, and even ruling out malaria will help to shift the focus to other possible causes.

Parasitological diagnosis by microscopy or RDT is recommended by the World Health Organization on all suspected malaria cases whenever either method is accessible [8]. Polymerase chain reaction (PCR) offers a lower limit of detection. Nested PCR limits of 0.4 and 0.05 parasites/μL have been reported, but it is barely applicable for routine diagnosis in endemic malaria fields due to its technical and economical requirements [9–11]. While these three methods are useful and bear different advantages to fit varied settings, none combines the accuracy of the nested PCR and field friendliness of the RDT.

In 2000, loop-mediated isothermal amplification (LAMP) became available as a novel DNA amplification and detection tool that was reported to operate at a sensitivity similar to that of PCR [12]. This amplification method uses four primers specifically designed to allow formation of a loop-structured complement of the target DNA by strand displacement. Such loop-structured DNA are later amplified at a stable temperature (~ 65 °C) and detected by the presence of magnesium pyrophosphate (by-product of DNA amplification) using a turbidimeter or calcein (released from its quenched state during amplification) fluorescence under ultraviolet light excitation.

Efforts to produce more accurate and field-friendly malaria diagnostic methods has given birth to several assays based on the LAMP [13–17]. Among them, the Loopamp™ Malaria Pan/Pf Detection Kit (Eiken Chemical, Tokyo, Japan) is the first LAMP kit for the detection of malaria parasites to be commercialized in field-friendly packaging containing tubes filled with reagents stable at room temperature. The Loopamp kit includes two types of reaction tubes: one with primers targeting the DNA sequence of the common *Plasmodium* genus (Pan tube), and the other specifically targeting that of *P. falciparum* (Pf tube). Thus, it allows the diagnosis of malaria and detects whether the causal agent(s) involve *P. falciparum* or other *Plasmodium* species. In parallel, a simple DNA extraction method, the procedure for ultra rapid extraction (PURE, Eiken Chemical), which produces DNA solution suitable for the LAMP reaction without the use of a centrifuge, has also been supplied. A study previously reported the Loopamp kit to be accurate for the detection of plasmodial DNA from dried blood samples on filter paper coupled with a conventional DNA extraction method [18]. In the present study, the accuracy of Loopamp malaria detection using DNA extracted from dried blood samples by the PURE method was evaluated in comparison to standard diagnosis by nested PCR, microscopy, and RDT. Hereafter, the term PURE–LAMP is used to refer to the combination of the PURE and Loopamp methods.

## Methods

### Blood samples

The samples included in this study were collected by venipuncture from patients who consulted the Center Hospital of the National Center for Global Health and Medicine (NCGM, Tokyo). The diagnosis of malaria was achieved with a combination of microscopic observation, RDT and nested PCR with these patients’ blood samples. To conduct PURE–LAMP, dried blood samples stored at room temperature on paper of a single-well preservation plate (WATSON, Tokyo, Japan) were used, and the results were retrospectively compared. One hundred and seventeen samples diagnosed in the period from July 2011 through December 2016 were included. The storage length before performing PURE–LAMP varied between the samples: roughly, the shortest period was 2 weeks and the longest was 5 years (Additional file 1).

### Microscopic diagnosis

The microscopic diagnosis was conducted by expert microscopists on light microscopes with Giemsa-stained thin blood smears. Malaria-negative samples were declared after counting ≥ 200,000 erythrocytes.

### RDT

RDT were conducted using BinaxNow^®^ Malaria (Alere Inc., Waltham, MA, USA). The test includes T1 and T2 bands. The T1 band contains the antibody for detection of a *P. falciparum*-specific HRP2 antigen, whereas the T2 band contains the antibody for detection of a *Plasmodium* common aldolase antigen. Pale bands were counted as positive.

### DNA extraction from patient blood samples

To obtain the template DNA used in the PCR reactions, several methods indicated below were utilized. In many cases, DNA was extracted from 200 µL of fresh blood or 100 µL of frozen RBC concentrate using a QIAamp^®^ DNA Mini Kit (QIAGEN, Hilden, Germany). An automated DNA extraction system, the Maxwell RSC Instrument (Promega, Madison, WI, USA), was also used. With this instrument, DNA samples were extracted from 200 µL of fresh blood or 100 µL of frozen RBC concentrate with a Maxwell RSC Blood DNA Kit (Promega), and after purification steps, DNA samples were eluted in 50 or 100 µL of elution buffer. In other cases, DNA were extracted from 3 dried blood spots (DBS) of ϕ 3 mm using a Maxwell RSC DNA FFPE Kit (Promega) with the automated Maxwell RSC Instrument, and DNA was eluted in 50 µL of buffer.

### Nested PCR

Two protocols were used for the nested PCR [19, 20]. In both protocols, the first PCR was conducted with primers targeting the universal partial sequences of the 18S ribosomal RNA (rRNA) gene of the *Plasmodium* genus. For the second PCR, dilutions of the first PCR products were used as templates along with a species-specific primer for each human Plasmodium species per tube.

### Sequencing analysis

Samples diagnosed as *Plasmodium ovale* were further defined into subspecies by sequencing of 365 nucleotides of the 18S rRNA gene. A nested PCR was designed for amplification of *P. ovale*-specific sequences. For the first PCR, 2 µL of template DNA eluted from the patient’s blood was added into 25 µL of reaction mix; a forward primer, F1 (5′-CTGGTGCCAGCAGCCGCGGTA-3′) and a reverse primer, R1 (5′-ATGAGAAATCAAAGTCTTTGGGTTC-3′), which are targeting a segment of ~ 660 bp of the *P. ovale* universal 18S rRNA gene, were used. After 1000-fold dilution of the first PCR product, 2 µL was added as template in 20 µL of the second PCR reaction mix with an inner forward primer, F2 (5′-CTGCGTTTGAATACTACAGCATGGA-3′), and the same reverse primer, R1. PrimeSTAR GXL DNA Polymerase (TaKaRa Bio Inc., Kusatsu, Japan) was used for both PCR reactions. After sequencing of the secondary amplicons, the subspecies were determined by sequence homology with the same genes of *P. ovale* subspecies registered in the GenBank sequence database (accession numbers AB182489 and AB182490 for *P. o. curtisi* and AB182491, AB182492, and AB182493 for *P. o. wallikeri*).

### PURE DNA extraction and LAMP reaction

DNA was extracted using the Loopamp™ PURE DNA Extraction Kit (Eiken Chemical) according to the manufacturer’s supplied protocol. Briefly, 3 DBS of ϕ 3 mm were punched into the heating tube containing extraction buffer, mixed by shaking and placed in a heating block (Loopamp™ LF-160, Eiken Chemical) at 75 °C for 5 min. After a rough powder purification of the lysate, the DNA-containing solution was eluted through an injection cap.

The LAMP reactions with the Loopamp™ MALARIA Pan/Pf Detection Kit were performed using 30 µL of the PURE-extracted DNA placed on a Loopamp™ LF-160 reaction block at 65 °C for 40 min followed by 5 min of incubation at 80 °C for enzyme inactivation. Each run was loaded with negative and positive controls, which were supplied in the kit. The results were evaluated by observation of fluorescence under excitation with an ultraviolet lamp incorporated into the same apparatus.

### Limit of detection

The PURE–LAMP limit of detection was analysed using infected human blood of known parasitaemia. Cultured *P. falciparum* strain FCR3 were synchronized with 5% d-sorbitol, and 40-h post-synchronization ring stage-infected RBCs were harvested, observed under 1000× light microscopy to determine parasitaemia, and dissolved in human whole blood to reach a concentration of 1000 parasites/μL. Series of 10× dilutions were prepared until the concentration reached 10^−3^ parasites/μL. A part of these blood samples was seeded on filter paper on the same day, and 30 μL was used to conduct the PURE–LAMP. After 3 months of storage at room temperature, the PURE–LAMP was repeated using 3 DBS of ϕ 3 mm. The PURE–LAMP could sometimes detect up to 0.1 parasite/μL in fresh blood but always 1 parasite/μL. The limit of detection of 1 parasite/μL was sustained with the DBS.

### Statistical analysis

Sensitivity [true positive/(true positive + false negative)] and specificity [true negative/(true negative + false positive)] with their exact 95% confidence interval were estimated using nested PCR as the gold-standard reference. Stata ver. 14.2 (StataCorp, College Station, TX, USA) was used for statistical analysis.

## Results

### Overview of the clinical samples used in this study

One hundred and seventeen samples were retrospectively analysed. A range of 7–19 positive cases was diagnosed per year (Additional file 1). The places of travel included countries in Africa, South America, Asia, and the West-Pacific region. Africa (66.66%) was the most prevalent region of provenance reported among the subjects, with histories of travel from Uganda (8 cases/10 samples) on top for number of positives. India was the most common country of provenance (2 cases/11 samples).

**Fig. 1:**
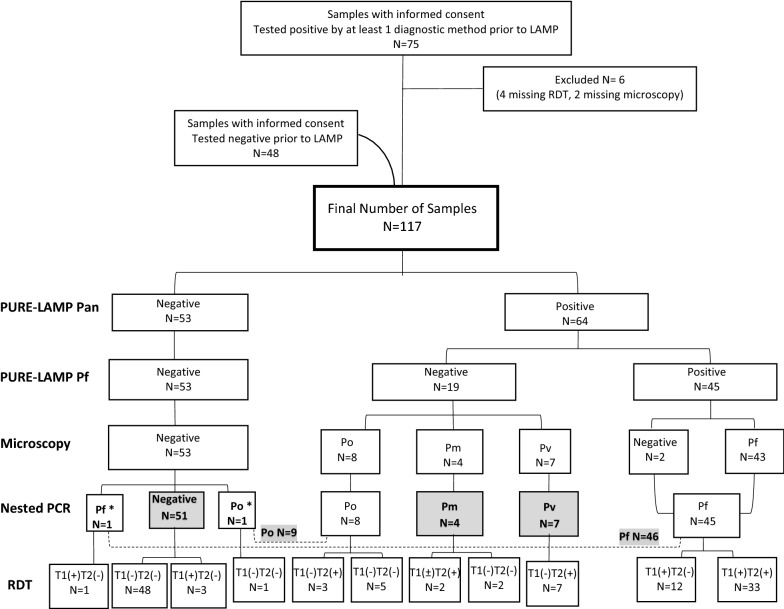
Flow chart of the study. Results of the PURE–LAMP Pan (tubes for diagnosis of *Plasmodium genus*), PURE–LAMP Pf (for diagnosis of *P. falciparum*), microscopy, nested PCR, and rapid diagnostic tests (RDT) are shown. Discrepant samples between PURE–LAMP and nested PCR are marked by an asterisk. The final number of cases per diagnosis type is highlighted. PURE–LAMP: procedure for ultra rapid extraction—loop-mediated isothermal amplification; Pf: *Plasmodium falciparum*; Po: *P. ovale*; Pv: *P. vivax*; Pm: *P. malariae*; DBS: dried blood spots

There were 46 (39.32%) cases of *P. falciparum*, 9 (7.69%) of *P. ovale*, 7 (5.98%) of *Plasmodium vivax*, 4 (3.42%) of *Plasmodium malariae* and 51 (43.59%) negative samples included in this study (Fig. 1). Two *P. ovale* cases classified as *P. o. wallikeri* and 6 cases classified as *P. o. curtisi*. One *P. ovale* sample could not be sequenced due to an extremely low amount of DNA.

### Comparison of PURE–LAMP and nested PCR

The results of the PURE–LAMP and the nested PCR were considerably correlated. The Pan tubes of the PURE–LAMP correctly identified 64 [Pan(+)] of 66 positive samples and all 51 [Pan(−)] negative samples. Among the 64 Pan(+) samples, 45 were also positive by the *P. falciparum*-specific tubes [Pf(+)]; these were 45 of 46 *P. falciparum* samples. No samples showed a result of Pan(−)Pf(+) (Fig. 1; Tables 1, 2).

**Table 1:** Sensitivity and specificity of PURE–LAMP, microscopy observation, and RDT compared to nested PCR

Test	Sensitivity [95% CI]	Specificity [95% CI]
PURE–LAMP Pan	96.96% [89.47–99.63]	100%
PURE–LAMP Pf	97.82% [88.47–99.94]	100%
Microscopy	93.93% [85.20–98.32]	100%
RDT	T1: 100% T2: 68.18% [55.56–79.11]	T1: 92.95% [84.32–97.67] T2: 100%

*PURE–LAMP* procedure for ultra rapid extraction-loop-mediated isothermal amplification, *RDT* rapid diagnostic test, *PCR* polymerase chain reaction, *CI* confidence interval, *Pan Plasmodium* genus, *Pf P. falciparum*, *T1* band of the rapid test containing the antibody for detection of a *Plasmodium falciparum*-specific HRP2 antigen, *T2* band containing the antibody for detection of a *Plasmodium* common aldolase antigen

**Table 2:** Detailed information of samples with discrepant results between PURE–LAMP and nested PCR

Sample no.^a^	RDT	Microscopy	PURE–LAMP Pan	PURE–LAMP Pf	Nested PCR
12	Negative T1(−)T2(−)	Negative	Negative	Negative	Po
78	Pf T1(+)T2(−)	Negative	Negative	Negative	Pf

*PURE–LAMP* procedure for ultra rapid extraction-loop-mediated isothermal amplification, *Pan Plasmodium* genus, *Pf P. falciparum*, *PCR* polymerase chain reaction, *RDT* rapid diagnostic test, *T1* band of the rapid test containing the antibody for detection of a *Plasmodium falciparum*-specific HRP2 antigen, *T2* band containing the antibody for detection of a *Plasmodium* common aldolase antigen

^a^Sample numbering is according to the list of all samples in the Additional file 1

### Comparison of PURE–LAMP and microscopic observations

All negatives by PURE–LAMP were also negative by microscopy. Only 2 positive samples by PURE–LAMP [Pan(+)Pf(+)], which were in conformity with the *P. falciparum* diagnosis by the nested PCR, were misdiagnosed by microscopy as negative. Parasitaemia by microscopy varied within a countable range from 0.001 to 21.7% and at uncountable level when 1 gametocyte is observed for over 200,000 erythrocytes (Additional file 1).

### Comparison of PURE–LAMP and RDT

The HRP2 band (T1) correctly detected all *P. falciparum* samples. Three of the PURE–LAMP negatives showed false positive [T1(+), PCR(−)]. All 7 *P. vivax* samples were detected by the T2 band, also were 3 of 9 *P. ovale* cases, 2 of 4 *P. malariae* cases and 33 of the 46 *P. falciparum* cases. The 2 *P. malariae* single infections were detected by the RDT indicating *P. falciparum* or mixed infection [T1(+)T2(+)]; and by the PURE–LAMP as Pan(+)Pf(−). In general, 8 positives non-*P. falciparum* samples were missed by the RDT while PURE–LAMP missed 1 *P. falciparum* and 1 *P. ovale* samples only.

## Discussion

This study examined the sensitivity of PURE–LAMP with archived clinical blood samples stored on dried filter paper in comparison to other standard methods. The results suggested a sensitivity higher than that of microscopy, around 97% of the nested PCR and a specificity of 1. Such sensitivity was expected based on previous evaluations of the Loopamp kit with other DNA extraction methods [16–18, 21, 22]. The 2 false-negative samples by PURE–LAMP in this study came from patients with negative microscopic observation and a history of recent anti-malarial drug use. Previous investigations showed the PURE–LAMP limit of detection to range between 0.33 and 3.33 parasites/μL using dried materials in which cultured *P. falciparum* parasites were mixed into healthy human blood [22], whereas Aydin-Schmidt et al. [18] reported a limit of ≤ 2 parasites/μL for the Loopamp kit using DNA extracted with Chelex (Bio-Rad, Hercules, CA, USA) from dried blood spots. As a limit of 1 parasite/μL was found with the method studied in this paper, it seems that both discrepant samples had parasitaemia under the Loopamp kit limit of detection or remaining DNA of dead parasites. The study by Aydin-Schmidt et al. [18] showed the accuracy of the Loopamp kit to detect malaria parasites at low density from dried samples on filter paper. The present study supported this finding and revealed that the use of the new PURE DNA extraction method reached similar results. Although the present PURE–LAMP analysis was conducted retrospectively, it also corroborates the usefulness of the method for the diagnosis of imported malaria in travellers as reported previously [16]. As exposed earlier, microscopy allows us more than dichotomous characterization of samples. However, the difference observed in sensitivity between PURE–LAMP and microscopy in the present study is expected to be wider in the field, where the PURE–LAMP sensitivity should be maintained, but the sensitivity of microscopy is expected to be lower at times [17, 21, 23].

This study included parasites of multiple provenances, with all 4 human transmissible species including both *P. ovale* subspecies that could be seen as an advantage however the modest sample size constituted a limitation (Additional file 1). No case of *Plasmodium knowlesi* was included: however, it can also be detected by PURE–LAMP (Kawai, unpublished data).

The LAMP technique has been adapted for use in the diagnosis of different infectious diseases with notoriously high burdens, including tuberculosis and malaria, for which high sensitivity, specificity, affordability, and applicability to the tropical field are desired [24]. Among the advantages of this kit compared to PCR are that it requires less investment, staff training, and operating time. Results can be ready within 1.5 h from punching DBS through DNA extraction and recording the results. A common disadvantage is the need for power during heating and reaction time.

## Conclusion

Filter paper is already a well-known field support method as it allows the collection, transfer, and storage of samples without a cold chamber. Considering the designed-in field-friendliness of this PURE–LAMP combination method, use of this system with dried blood samples will allow maximum exploitation of this characteristic while still performing accurately.

Cost-effectiveness is beyond the scope of this paper, but technically this system is predicted to be extendable to the field, while it is effective for malaria diagnosis in travellers.

## Additional file

**Additional file 1.** List of samples analysed: Combination of PURE-DNA extraction and LAMP-DNA amplification methods for accurate malaria diagnosis on dried blood spots. PURE-LAMP: Procedure for ultra rapid extraction–loop-mediated isothermal amplification; Pan: *Plasmodium* genus; Pf: *Plasmodium falciparum*; Pv: *P. vivax*; Pm: *P. malariae*; Po: *P. ovale*; Poc: *P. o. curtisi*; Pow: *P. o. wallikeri*; RBC: red blood cells; T1: band of the rapid test containing the antibody for detection of a *Plasmodium falciparum*-specific HRP2 antigen; T2: band containing the antibody for detection of a *Plasmodium* common aldolase antigen. +: positive; −: negative; ±: positive (pale band). *DNA extracted from 200 μL of fresh blood or 100 μL of frozen RBC concentrate, nested PCR method 1 [19]. **DNA extracted from 200 μL of fresh blood or 100 μL of frozen RBC concentrate, nested PCR method 2 [20]. ***DNA extracted from 3 dried blood spots of ϕ 3 mm, nested PCR method 2 [20].

## Abbreviations

LAMP: loop-mediated isothermal amplification
PCR: polymerase chain reaction
Pf: *P. falciparum*
Pm: *P. malariae*
Po: *P. ovale*
PURE: procedure for ultra rapid extraction
*Pv*: *P. vivax*
RDT: rapid diagnostic tests
rRNA: small subunit ribosomal RNA
DBS: dried blood spot
RBC: red blood cells
