# Supplementary material for: Combination of PURE-DNA extraction and LAMP-DNA amplification methods for accurate malaria diagnosis on dried blood spots
Source: Malar J. 2018 Oct 22;17:373. doi: 10.1186/s12936-018-2527-7 (PMC6196555; doi:10.1186/s12936-018-2527-7)
Supplement: Supplementary file 1 — Additional file 1. List of samples analysed: Combination of PURE-DNA extraction and LAMP-DNA amplification methods for accurate malaria diagnosis on dried blood spots. PURE-LAMP: Procedure for ultra rapid extraction–loop-mediated isothermal amplification; Pan: Plasmodium genus; Pf: Plasmodium falciparum; Pv: P. vivax; Pm: P. malariae; Po: P. ovale; Poc: P. o. curtisi; Pow: P. o. wallikeri; RBC: red blood cells; T1: band of the rapid test containing the antibody for detection of a Plasmodium falciparum-specific HRP2 antigen; T2: band containing the antibody for detection of a Plasmodium common aldolase antigen. +: positive; −: negative; ±: positive (pale band). *DNA extracted from 200 μL of fresh blood or 100 μL of frozen RBC concentrate, nested PCR method 1 [19]. **DNA extracted from 200 μL of fresh blood or 100 μL of frozen RBC concentrate, nested PCR method 2 [20]. ***DNA extracted from 3 dried blood spots of ϕ 3 mm, nested PCR method 2 [20]. [file 12936_2018_2527_MOESM1_ESM.docx]

**Additional file 1**

**List of samples analysed:** **Combination of PURE-DNA extraction and LAMP-DNA amplification methods for accurate malaria diagnosis on dried blood spots**

| **Sample number** | **Date** | **Provenance**  **(refer travel from)** | **Rapid diagnostic test** | **Microscopic parasitemia** | **PURE-LAMP Pan** | **PURE-LAMP Pf** | **Nested PCR** |
| --- | --- | --- | --- | --- | --- | --- | --- |
| 1 | Jul-11 | Ghana | T1(+)T2(±) | Pf 0.13% | Positive | Positive | Pf*** |
| 2 | Jul-11 | Pakistan | T1(-)T2(+) | Pv 0.26% | Positive | Negative | Pv* |
| 3 | Jul-11 | Togo | T1(+)T2(±) | Pf 0.33% | Positive | Positive | Pf*** |
| 4 | Aug-11 | PNG | T1(-)T2(±) | Pv 0.021% | Positive | Negative | Pv* |
| 5 | Sep-11 | South Sudan, Zambia | T1(+)T2(+) | Pf 4.8% | Positive | Positive | Pf*** |
| 6 | Oct-11 | Burkina Faso | T1(+)T2(±） | Pf 0.08% | Positive | Positive | Pf*** |
| 7 | Dec-11 | Mozambique | T1(±)T2(+) | Pm 0.027% | Positive | Negative | Pm* |
| 8 | Jan-12 | West African Countries | T1(+)T2(-) | Pf (Only one dead gametocyte) | Positive | Positive | Pf*** |
| 9 | Feb-12 | Indonesia | T1(+)T2(+) | Pf 13% | Positive | Positive | Pf*** |
| 10 | Mar-12 | Ivory Coast | T1(+)T2(-) | Pf 1/17500 RBC | Positive | Positive | Pf*** |
| 11 | Mar-12 | Zambia, Congo, Senegal, Sudan | T1(±)T2(-) | 0/250000 RBC | Positive | Positive | Pf* |
| 12 | Apr-12 | Kenya, Thailand, Malaysia | T1(-)T2(-) | Negative | Negative | Negative | Po* |
| 13 | Apr-12 | Uganda | T1(-)T2(-) | Po 0.042% | Positive | Negative | Poc* |
| 14 | Jul-12 | Pakistan | T1(-)T2(+) | Pv 0.5% | Positive | Negative | Pv* |
| 15 | Aug-12 | Uganda | T1(+)T2(+) | Pf 7.3% | Positive | Positive | Pf*** |
| 16 | Aug-12 | Nigeria | T1(+)T2(+) | Pf 0.53% | Positive | Positive | Pf*** |
| 17 | Sep-12 | Nigeria | T1(+)T2(+) | Pf 0.61% | Positive | Positive | Pf*** |
| 18 | Sep-12 | Ghana | T1(+)T2(+) | Pf 0.5% | Positive | Positive | Pf*** |
| 19 | Nov-12 | Uganda, Rwanda | T1(+)T2(+) | Pf 0.1% | Positive | Positive | Pf*** |
| 20 | Nov-12 | Angola | T1(-)T2(-) | Negative | Negative | Negative | Negative** |
| 21 | Dec-12 | Nigeria | T1(+)T2(±） | Pf 0.23% | Positive | Positive | Pf*** |
| 22 | Dec-12 | Myanmer | T1(-)T2(-) | Negative | Negative | Negative | Negative** |
| 23 | Dec-12 | PNG | T1(-)T2(-) | Negative | Negative | Negative | Negative** |
| 24 | Dec-12 | Ghana | T1(-)T2(-) | Negative | Negative | Negative | Negative** |
| 25 | Dec-12 | Cambodia | T1(-)T2(-) | Negative | Negative | Negative | Negative** |
| 26 | Dec-12 | Philippine | T1(-)T2(-) | Negative | Negative | Negative | Negative** |
| 27 | Jan-13 | Uganda | T1(+)T2(-) | Pf 0.006% | Positive | Positive | Pf*** |
| 28 | Jan-13 | Zambia | T1(-)T2(-) | Negative | Negative | Negative | Negative** |
| 29 | Jan-13 | Brazil | T1(-)T2(-) | Negative | Negative | Negative | Negative** |
| 30 | Feb-13 | India, Zimbabwe | T1(-)T2(-) | Negative | Negative | Negative | Negative** |
| 31 | Feb-13 | Kenya | T1(-)T2(-) | Negative | Negative | Negative | Negative** |
| 32 | Mar-13 | Uganda | T1(-)T2(-) | Negative | Negative | Negative | Negative** |
| 33 | Mar-13 | Etiopia | T1(-)T2(-) | Negative | Negative | Negative | Negative** |
| 34 | Mar-13 | Cambodia | T1(-)T2(-) | Negative | Negative | Negative | Negative** |
| 35 | Mar-13 | India | T1(-)T2(-) | Negative | Negative | Negative | Negative** |
| 36 | Mar-13 | India | T1(-)T2(-) | Negative | Negative | Negative | Negative** |
| 37 | Mar-13 | Sierra Leone, Guinea | T1(+)T2(±) | Pf 0.003% | Positive | Positive | Pf*** |
| 38 | Mar-13 | Indonesia | T1(-)T2(-) | Negative | Negative | Negative | Negative** |
| 39 | Apr-13 | Sri Lanka | T1(-)T2(-) | Negative | Negative | Negative | Negative** |
| 40 | Apr-13 | Uganda | T1(-)T2(-) | Po or Pv 0.014% | Positive | Negative | Po* |
| 41 | May-13 | Ghana | T1(-)T2(-) | Negative | Negative | Negative | Negative** |
| 42 | May-13 | Indonesia | T1(-)T2(-) | Negative | Negative | Negative | Negative** |
| 43 | May-13 | Tanzania | T1(+)T2(+) | Pf 3.5% | Positive | Positive | Pf*** |
| 44 | Jun-13 | Kenya | T1(-)T2(-) | Negative | Negative | Negative | Negative** |
| 45 | Jun-13 | Senegal | T1(-)T2(-) | Negative | Negative | Negative | Negative** |
| 46 | Jul-13 | Guinea, Senegal | T1(-)T2(-) | Negative | Negative | Negative | Negative** |
| 47 | Jul-13 | Malaysia | T1(-)T2(-) | Negative | Negative | Negative | Negative** |
| 48 | Jul-13 | Tanzania | T1(-)T2(-) | Negative | Negative | Negative | Negative** |
| 49 | Jul-13 | India | T1(-)T2(-) | Negative | Negative | Negative | Negative** |
| 50 | Jul-13 | Indonesia | T1(-)T2(-) | Negative | Negative | Negative | Negative** |
| 51 | Aug-13 | India | T1(-)T2(-) | Negative | Negative | Negative | Negative** |
| 52 | Aug-13 | Philippine | T1(-)T2(-) | Negative | Negative | Negative | Negative** |
| 53 | Sep-13 | Senegal | T1(-)T2(-) | Negative | Negative | Negative | Negative** |
| 54 | Sep-13 | Zimbabwe, Botswana | T1(-)T2(-) | Negative | Negative | Negative | Negative** |
| 55 | Oct-13 | Congo | T1(+)T2(-) | 0/400000 RBC | Negative | Negative | Negative*** |
| 56 | Nov-13 | Benin | T1(+)T2(±) | Pf 0.08% | Positive | Positive | Pf*** |
| 57 | Nov-13 | South Sudan | T1(-)T2(-) | Pm 4/241131 RBC | Positive | Negative | Pm* |
| 58 | Nov-13 | Cambodia | T1(-)T2(-) | Negative | Negative | Negative | Negative** |
| 59 | Dec-13 | Angola | T1(-)T2(-) | ? 4/252930 | Positive | Negative | Pm* |
| 60 | Jan-14 | Tanzania | T1(-)T2(-) | Negative | Negative | Negative | Negative** |
| 61 | Feb-14 | Cameroon | T1(+)T2(-) | Pf 0.04% | Positive | Positive | Pf*** |
| 62 | Mar-14 | India | T1(-)T2(-) | Negative | Negative | Negative | Negative** |
| 63 | Mar-14 | Guinea | T1(+)T2(+) | Pf 2.1% | Positive | Positive | Pf*** |
| 64 | Mar-14 | Zambia | T1(-)T2(-) | Po? | Positive | Negative | Pow* |
| 65 | Apr-14 | Uganda | T1(+)T2(-) | Pf 9/256706 RBC | Positive | Positive | Pf*** |
| 66 | Apr-14 | Kenya | T1(+)T2(+) | Pf 21.7% | Positive | Positive | Pf*** |
| 67 | Apr-14 | Venezuela | T1(+)T2(±) | Pf 0.24% | Positive | Positive | Pf*** |
| 68 | Apr-14 | Thailand, Cambodia, Laos, Vietnam | T1(-)T2(-) | Negative | Negative | Negative | Negative** |
| 69 | May-14 | Vietnam, Cambodia | T1(-)T2(-) | Negative | Negative | Negative | Negative** |
| 70 | May-14 | Morocco, Senegal, Mauritania, Gambia | T1(-)T2(-) | Negative | Negative | Negative | Negative** |
| 71 | May-14 | Singapore, Myanmar | T1(-)T2(-) | Negative | Negative | Negative | Negative** |
| 72 | May-14 | Vietnam | T1(-)T2(-) | Negative | Negative | Negative | Negative** |
| 73 | May-14 | Nigeria, Benin | T1(+)T2(+) | Pf 0.31% | Positive | Positive | Pf*** |
| 74 | Jun-14 | Indonesia | T1(-)T2(-) | Negative | Negative | Negative | Negative** |
| 75 | Jun-14 | Sierra Leone | T1(+)T2(+) | Pf 0.64% | Positive | Positive | Pf*** |
| 76 | Jun-14 | India | T1(-)T2(-) | Negative | Negative | Negative | Negative** |
| 77 | Jun-14 | Mozambique | T1(-)T2(-) | Negative | Negative | Negative | Negative** |
| 78 | Jun-14 | Sierra Leone | T1(+)T2(-) | 0/347271 | Negative | Negative | Pf*** |
| 79 | Jun-14 | India | T1(-)T2(-) | Negative | Negative | Negative | Negative** |
| 80 | Jul-14 | Liberia | T1(-)T2(-) | Negative | Negative | Negative | Negative** |
| 81 | Jul-14 | Sudan | T1(-)T2(-) | Negative | Negative | Negative | Negative** |
| 82 | Jul-14 | Cameroon | T1(-)T2(±) | Po 0.072% | Positive | Negative | Poc** |
| 83 | Jul-14 | Guinea | T1(+)T2(+) | Pf 15.52% | Positive | Positive | Pf** |
| 84 | Jul-14 | Indonesia | T1(-)T2(-) | Negative | Negative | Negative | Negative** |
| 85 | Aug-14 | Bangladesh | T1(-)T2(-) | Negative | Negative | Negative | Negative** |
| 86 | Sep-14 | Vietnam | T1(-)T2(-) | Negative | Negative | Negative | Negative** |
| 87 | Sep-14 | PNG | T1(-)T2(±) | Pv 0.24% | Positive | Negative | Pv** |
| 88 | Sep-14 | India, Nepal | T1(-)T2(-) | Negative | Negative | Negative | Negative** |
| 89 | Apr-15 | Congo | T1(+)T2(+) | Pf 0.22% | Positive | Positive | Pf** |
| 90 | Apr-15 | Cameroon | T1(+)T2(+) | Pf 0.44% | Positive | Positive | Pf** |
| 91 | May-15 | Uganda | T1(+)T2(+) | Pf 0.725% | Positive | Positive | Pf** |
| 92 | Jun-15 | Uganda | T1(+)T2(-) | Pf 0.023% | Positive | Positive | Pf** |
| 93 | Jun-15 | PNG | T1(+)T2(+) | Pf 0.21% | Positive | Positive | Pf** |
| 94 | Jun-15 | Ivory Coast | T1(+)T2(+) | Pf 1.78% | Positive | Positive | Pf** |
| 95 | Aug-15 | India | T1(-)T2(+) | Pv 0.16% | Positive | Negative | Pv** |
| 96 | Aug-15 | Ivory Coast | T1(±)T2(+) | Pm 0.125% | Positive | Negative | Pm** |
| 97 | Feb-16 | Cameroon | T1(+)T2(-) | Pv 0.0094% | Positive | Positive | Pf** |
| 98 | Feb-16 | Zambia | T1(+)T2(-) | Pf 0.34% | Positive | Positive | Pf** |
| 99 | Mar-16 | Conco, Chile | T1(+)T2(+) | Pf 0.068% | Positive | Positive | Pf** |
| 100 | Apr-16 | Zambia | T1(-)T2(±) | Po 0.028% | Positive | Negative | Poc** |
| 101 | Apr-16 | Ivory Coast | T1(+)T2(-) | Pf 1.052% | Positive | Positive | Pf** |
| 102 | Apr-16 | Ghana | T1(-)T2(±) | Po 0.038% | Positive | Negative | Poc** |
| 103 | Apr-16 | Cameroon | T1(+)T2(+) | Pf 0.011% | Positive | Positive | Pf** |
| 104 | Apr-16 | Ivory Coast | T1(+)T2(+) | Pf 4.5% | Positive | Positive | Pf** |
| 105 | Apr-16 | Ivory Coast | T1(+)T2(+) | Pf 2.1% | Positive | Positive | Pf** |
| 106 | May-16 | Angola | T1(+)T2(-) | Negative | Negative | Negative | Negative** |
| 107 | May-16 | Angola | T1(-)T2(-) | Po 0.001% | Positive | Negative | Pow** |
| 108 | May-16 | Angola | T1(+)T2(+) | Pf 0.15% | Positive | Positive | Pf** |
| 109 | Jul-16 | India | T1(-)T2(+) | Pv 0.2% | Positive | Negative | Pv** |
| 110 | Jul-16 | Ghana | T1(+)T2(+) | Pf 0,18% | Positive | Positive | Pf** |
| 111 | Jul-16 | African countries | T1(+)T2(-) | Pf 0.016% | Positive | Positive | Pf** |
| 112 | Aug-16 | Rwanda | T1(+)T2(+) | Pf 1.5% | Positive | Positive | Pf** |
| 113 | Aug-16 | Ivory Coast | T1(-)T2(-) | Po 0.05% | Positive | Negative | Poc** |
| 114 | Oct-16 | Nigeria | T1(+)T2(+) | Pf 0.058% | Positive | Positive | Pf** |
| 115 | Nov-16 | PNG | T1(-)T2(+) | Pv 0.11% | Positive | Negative | Pv** |
| 116 | Dec-16 | Uganda | T1(+)T2(-) | Negative | Negative | Negative | Negative** |
| 117 | Dec-16 | African and Asian countries | T1(+)T2(-) | Negative | Positive | Positive | Pf** |

PURE-LAMP: Procedure for ultra rapid extraction–loop-mediated isothermal amplification; Pan: *Plasmodium* genus; +: positive; −: negative; ±: positive (pale band); Pf: *Plasmodium falciparum*; Pf: *Plasmodium falciparum*; Pv: *P. vivax*; Pm: *P. malariae*; Po: *P. ovale*; Poc: *P. ovale curtisi*; Pow: *P. ovale wallikeri*; RBC: red blood cells.

*DNA extracted from 200 μL of fresh blood or 100 μL of frozen RBC concentrate, nested PCR method 1 [19].

**DNA extracted from 200 μL of fresh blood or 100 μL of frozen RBC concentrate, nested PCR method 2 [20].

***DNA extracted from 3 dried blood spots of φ 3 mm, nested PCR method 2 [20].
